# Going Underground: How Perceived High-Performance Work Systems Influence Bootlegging Behavior? A Multi-Level Moderated Mediation Approach

**DOI:** 10.3390/bs14080657

**Published:** 2024-08-01

**Authors:** Asaad Salam Farooqi, Dian Song, Yishuai Yin, Yongzhi Yuan

**Affiliations:** 1 Dongwu Business School, Soochow University, Suzhou 215000, China; asfarooqi@stu.suda.edu.cn; 2 School of Political Science and Public Administration, Soochow University, Suzhou 215000, China; diansnow@suda.edu.cn (D.S.); yuanyongzhi@suda.edu.cn (Y.Y.)

**Keywords:** perceived HPWS, bootlegging behavior, willingness to take risks, creative self-efficacy, HRM system strength

## Abstract

This study applies the social information processing theory to investigate how perceived high-performance work systems (HPWS) influence bootlegging behavior. Additionally, it explores the potential mediating role of willingness to take risks and creative self-efficacy in the association between perceived HPWS and bootlegging behavior. In addition, this study examines how human resource management (HRM) system strength acts as a cross-level moderator in the connection between perceived HPWS and willingness to take risks and creative self-efficacy. Based on a sample of 399 respondents from 80 firms, the results indicate a positive connection between perceived HPWS and bootlegging behavior. Moreover, the results reveal that willingness to take risks and creative self-efficacy mediate the connection between perceived HPWS and bootlegging behavior. Findings also reveal that cross-level HRM system strength moderates the influence of perceived HPWS on willingness to take risks and creative self-efficacy. The study also highlights its theoretical contributions and practical implications and proposes avenues for future research.

## 1. Introduction

Strategic human resource management (SHRM) has been a research focus for over three decades and has recently prompted in-depth discussions [1]. Researchers investigated how high-involvement work systems and HPWS as bundles of HR practices assist firms in accomplishing goals. HPWS refers to a system of HR practices designed to enhance employees skills, commitment, and productivity in such a way that employees become a source of sustainable competitive advantage [2]. Researchers have demonstrated a double-edged sword effect of perceived HPWS, e.g., a positive and negative effect of perceived HPWS on various outcomes such as service encounter quality, innovation, employee engagement, organizational citizenship behavior, job burnout, and emotional exhaustion [3,4,5,6,7,8].

Although the importance of the double-edged sword effect of HPWS on various outcomes is well highlighted, limitations still exist in the theoretical, methodological, and contextual aspects of this research area, specifically regarding three unsolved issues.

First, the existing body of literature offers incongruent theoretical and empirical findings regarding the dual impact of perceived HPWS on top-down instructed outcomes for shaping and enhancing organizational effectiveness and performance [3,4,5,6,7,8]. According to our knowledge, there has not been enough theoretical advancement or empirical research to establish a connection between perceived HPWS and the phenomenon of “bootlegging”, which pertains to the bottom-up instructed outcome. Bootlegging behavior refers to working on secret ideas through motivated employees without having formal approval from higher authority to generate innovations that benefit the organization [9,10]. To confront this significant issue, we turn to the social information processing theory [11,12] with the primary objective of proposing a positive association of perceived HPWS with bootlegging behavior. Another reason to incorporate bootlegging behavior as an outcome is its importance, as bootlegging involves employees taking unconventional approaches to solve problems or improve processes. This leads to innovative solutions that may not have arisen through more structured means. Also, bootlegging represents a form of grassroots innovation where employees take the initiative to drive change from the bottom up. This results in quicker responses to emerging challenges and opportunities [10].

Second, the existing body of literature presents differing theoretical and empirical conclusions concerning various underlying mechanisms in the connection between perceived HPWS and several outcomes [7,13,14,15,16,17], but according to the best of our knowledge, there is a lack of theoretical development and empirical study exploring the relevant attitudes as underlying mechanisms in the association between perceived HPWS and bootlegging behavior. To deal with this pivotal concern, we employ the social information processing theory [11,12] with a secondary objective of proposing willingness to take risks and creative self-efficacy as mediators in the association between perceived HPWS and bootlegging behavior. Another reason to include willingness to take risks as a mediator is due to its importance, as bootlegging involves employees taking the initiative to innovate and experiment with new ideas or approaches. Thus, willingness to take risks is closely tied to bottom-up innovation because it requires individuals to step out of their comfort zones and try unconventional methods. Risk-takers exhibit strong problem-solving skills and are more adept at making decisions in uncertain situations, aligning with the requirements of HPWS. On the other hand, another compelling rationale for incorporating creative self-efficacy as a mediator is its significance, as bootlegging involves finding innovative solutions to problems or challenges that may not have conventional answers. Creative self-efficacy equips employees with the confidence to tackle such issues creatively and innovatively, which can align with the objectives of HPWS [18].

Third, previous researchers examined how various moderators affect perceived HPWS and different outcomes [6,8,14,15,16,19], but to date, no study has explored the effect of HRM system strength as a cross-level moderator in the association between perceived HPWS and willingness to take risks and creative self-efficacy. Following these logics, this study’s tertiary objective is to examine the cross-level moderating effect of HRM system strength in the relationship between perceived HPWS and willingness to take risks and creative self-efficacy.

**Figure 1:**
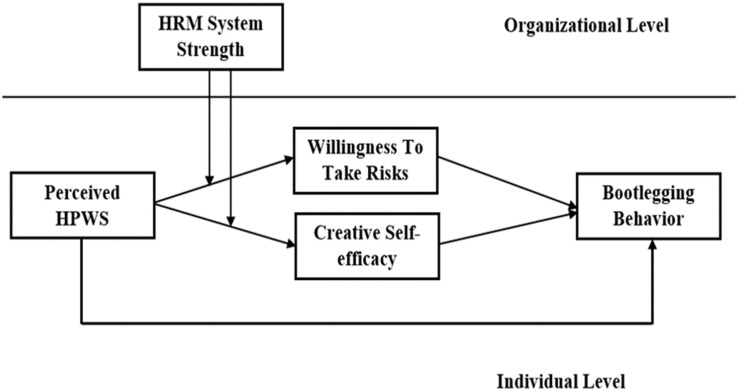
Theoretical model.

Social information processing theory posits that employees utilize informational cues for understanding and perceiving social information and the working environment that regulates their behaviors directly and indirectly through attitudes [11,12]. Behaviors, attitudes, and perceptions influenced by social information are cognitive outcomes that emerge through information processing [20]. Thus, social information processing theory underpins this study. This paper treats perceived HPWS as an employee’s perceived job characteristic, the social construction of reality; HRM system strength as work environment; willingness to take risks and creative self-efficacy as employee attitudes; and bootlegging behavior as behavior. Figure 1 shows the study’s theoretical model (see Figure 1).

## 2. Theory and Hypotheses

### 2.1. Social Information Processing Theory

In accordance with the tenets of the social information processing theory, employees use informational cues to understand and perceive social information and the work environment, which regulates their behavior directly and indirectly through attitudes [11,12]. Prior research has validated the social information processing theory for HPWS [21,22,23,24]. HPWS provides training and development programs to update employees’ knowledge and skills. Training employees in problem-solving, creative thinking, and innovative techniques equips them with the confidence and skills to exceed their job roles, which can lead to bootlegging behavior. When employees are empowered through HPWS to make decisions autonomously and take initiative, they often address issues and improve processes to generate bootlegging behavior. Additionally, HPWS include mechanisms such as rewards and bonuses that motivate employees to generate novel ideas or refine existing processes, further contributing to bootlegging behavior [10,25,26,27,28]. As HPWS helps employees accumulate knowledge through training and development, they are more inclined to embrace risk-taking, as they have confidence in their abilities to effectively tackle possible roadblocks. The implementation of HPWS fosters a feeling of ownership among employees in relation to their work and duties through practices such as involvement and empowerment. Employees who feel a strong sense of ownership are more inclined to assume responsibility for the risks inherent in their tasks or projects, ultimately contributing to the organization’s overall success. HPWS include performance-based incentives and rewards. Additionally, when employees perceive the possibility of receiving tangible rewards for their performance, they are motivated and are more inclined to take risks. Employees who are more willing to take risks show a greater sense of autonomy and initiative. They are inclined to try out new ideas or approaches, even if they do not follow formal procedures, because they believe it can result in positive outcomes, which leads to bootlegging behavior. Moreover, employees’ willingness to take risks motivates them to find different solutions to problems. They grab this opportunity to enhance existing processes or tackle problems that are affecting productivity or performance, even if it is not officially approved, resulting in bootlegging behavior [29,30,31,32]. HPWS generally focus on investing in programs for training and developing skills. These programs comprise creative problem-solving techniques and innovation-related skills that enhance employees’ confidence and belief in their abilities to perform creative work effectively. HPWS frequently includes employees in decision-making processes, encompassing decisions regarding work processes, goals, and strategies. When employees feel that their input is valued and considered during decision-making, they are more likely to have confidence in the value of their creative contributions and perform creative work efficiently and effectively. Additionally, HPWS frequently link recognition and reward to performance, encompassing creative problem-solving and innovation. Acknowledging and valuing employees’ creativity through rewards increases their creative self-efficacy. Employees harboring a robust belief in their creative capabilities tend to be more predisposed towards proactive initiatives aimed at problem-solving or capitalizing on opportunities for the betterment of the organization. They are more likely to think creatively, initiate change, explore and suggest novel concepts and ideas, experiment with new approaches, and provide creative solutions without formal approval or direction, resulting in bootlegging behavior [10,33,34].

HRM system strength communicates to employees about the value of creative self-efficacy and willingness to take risks in an organizational context. A robust HRM system strength in conjunction with perceived HPWS practices like training, supportive supervision, autonomy, involvement in decision-making, and rewards enhance employee motivation and self-confidence to show a willingness to take risks and also enhance belief to be creative [30,33,35,36].

### 2.2. Perceived HPWS and Bootlegging Behavior

Bootlegging behavior refers to working on secret ideas through motivated employees without having formal approval from higher authority to generate innovations that benefit the organization [9,10]. Employee bootlegging also refers to unscheduled activities within experimental units, such as research and development [37].

Following the social information processing theory, employees perceive social information, e.g., perceived HPWS, which influences behavior, e.g., bootlegging behavior [11,12,20]. For instance, HPWS encompass training and development programs that equip employees with the knowledge and skills that enhance their confidence and skills to go beyond their job roles, leading to bootlegging behavior [26,28,38]. HPWS often involves giving employees more autonomy and decision-making authority in their roles. Empowered employees may engage in addressing issues or improving processes without waiting for formal approval, as they believe in their ability to make decisions and effect positive change, leading to bootlegging behavior. Furthermore, HPWS offer monetary rewards and bonuses that motivate employees and provide them with a tangible incentive to put in extra effort to generate innovative ideas or improve existing processes without any formal approval to benefit the firm, leading to bootlegging behavior [10,25,27]. Based on the preceding arguments, we therefore propose:

**H1:**  *There is a positive relationship between perceived HPWS and bootlegging behavior.*

### 2.3. Mediating Role of Willingness to Take Risks

Willingness to take risks is defined as being willing to take potential risks to achieve beneficial outcomes for the organization, even if it has negative personal consequences [39].

Social information processing theory suggests that employees use cues from perceived HPWS to shape their attitudes and behaviors, e.g., willingness to take risks and bootlegging behavior [11,12,20]. For example, HPWS typically enhance employee knowledge and skills through training and development. When employees feel confident in their skills and knowledge, they may be more willing to take risks, knowing they have the necessary competencies to handle potential challenges. HPWS practice, e.g., involvement and empowerment, gives employees a sense of ownership over their work and responsibilities. Employees who feel a strong sense of ownership are more likely to take ownership of risks associated with their tasks or projects and can contribute to the organization’s success. HPWS often include performance-based incentives and rewards. Moreover, employees who see the potential for tangible rewards contingent on their performance may exhibit increased motivation to undertake risks that could lead to higher rewards [29,30].

Employees who are more willing to take risks exhibit a greater sense of autonomy and initiative. They are more likely to take the initiative to experiment with new ideas or approaches, even if they fall outside formal procedures, but believe that it can lead to positive outcomes, leading to bootlegging behavior. Moreover, willingness to take risks drives employees to seek alternative solutions to problems. They see an opportunity to improve existing processes or address issues that are hindering productivity or performance, even if they fall outside formal approval, leading to bootlegging behavior [30,32,37]. Based on the preceding arguments, we therefore propose:

**H2:**  *Willingness to take risks mediates the relationship between perceived HPWS and bootlegging behavior.*

### 2.4. Mediating Role of Creative Self-Efficacy

Creative self-efficacy pertains to an employee’s belief in their abilities to generate creative work through motivation and cognition [18].

Social information processing theory states that employees perceive social information, e.g., perceived HPWS, which influences employee attitudes, e.g., creative self-efficacy, and these attitudes further shape behaviors, e.g., bootlegging behavior [11,12,20]. For instance, HPWS usually focus on training and skill development initiatives, e.g., creative problem-solving techniques and innovation-related skills, which boost employees’ confidence and belief in their abilities to undertake creative work effectively. HPWS often involve employees in decision-making processes, including decisions related to work processes, goals, and strategies. When an employee’s input is valued and considered in decision-making, they are more likely to believe that their creative contributions are valuable and can undertake creative work efficiently and effectively. In addition, HPWS often tie recognition and rewards to performance, including creative problem-solving and innovation. Employees rewarded for their creative efforts are more likely to have higher creative self-efficacy as they see their creativity being acknowledged and valued [33,40].

Following social information processing theory, employees perceive social cues to develop attitudes, which turn into behavior [11,12,20]. Employees exhibiting elevated levels of creative self-efficacy tend to be more inclined to proactively address issues or capitalize on opportunities to benefit the firm. In turn, they are more inclined to think outside the box, initiate change, explore unconventional ideas, suggest new ideas and experiment with new approaches, and introduce creative solutions without waiting for formal approval or direction, leading to bootlegging behavior [10,34,41]. Based on the preceding arguments, we therefore propose:

**H3:**  *Creative self-efficacy mediates the relationship between perceived HPWS and bootlegging behavior.*

### 2.5. Cross-Level Moderating Effect of HRM System Strength

HRM system strength enables top management to communicate HRM policies with employees to promote shared perceptions at all levels [42]. A strong HRM system has three key traits: distinctiveness; consistency; and consensus. Distinctiveness means HR practices are visible, understandable, relevant, and legitimate to employees. Visibility means that employees are aware of HR practices and can easily access information about them. Relevance indicates that HR practices are aligned with the needs and priorities of employees and the organization. Understandability relates to the simplicity and clarity of HR practices and policies to avoid confusion or ambiguity. Legitimacy reflects the fairness, equity, and adherence to legal and ethical standards in HR practices. Consistency refers to the instrumentality, validity, and consistency of HRM messages. Instrumentality refers to the degree to which HRM messages are designed and communicated to achieve specific outcomes or objectives within the organization. Validity relates to the accuracy, truthfulness, and relevance of HRM messages. It signifies that the information conveyed is trustworthy and aligns with facts and reality. Within the context of HRM messages, consistency denotes the uniformity and cohesiveness of these messages across various communication channels and over time. Consensus refers to a high degree of agreement, alignment, and shared understanding among key stakeholders within an organization regarding HRM practices, policies, and strategies [42,43,44,45,46,47]. When employees view the HRM as distinct, consistent, and consensual throughout their firm, they recognize what the organization expects from them [43,44,45,46].

Individual perceptions determine how perceived HPWS and work environment (e.g., HRM system strength) affect willingness to take risks and creative self-efficacy [48].

Following the social information processing theory, employees understand and perceive the social information from their working environment to shape their perceptions, attitudes, and behaviors [11,12,20]. For instance, a robust HRM system effectively conveys to employees that the organization places importance on their ability to be creative and take risks. When managers focus on the elements, e.g., consistency, distinctiveness, and consensus in building a robust HRM system, employees develop a heightened awareness of HRM significance within the organization. This, in turn, facilitates a clearer understanding and perception of HRM messages. Consequently, it is anticipated that the strength of the HRM system will amplify the influence of perceived HPWS on both the willingness to take risks and creative self-efficacy. For example, supportive supervision, training and development, autonomy, involvement in decision-making, rewards, and information sharing in the presence of HRM system strength boost employees’ motivation, self-belief, and confidence to undertake creative work effectively and to show a willingness to take risks [30,33,35,36]. Based on the preceding arguments, we therefore propose:

**H4a:**  *Cross-level HRM system strength moderates the relationship between perceived HPWS and willingness to take risks. In the context of high-level HRM system strength, the association between perceived HPWS and willingness to take risks will be higher.*

**H4b:**  *Cross-level HRM system strength moderates the relationship between perceived HPWS and creative self-efficacy. In the context of high-level HRM system strength, the association between perceived HPWS and creative self-efficacy will be higher.*

## 3. Research Design

### 3.1. Data Collection and Sample

Data were collected from the manufacturing and services sectors of Pakistan. According to the International Standard Classification of All Economic Activities, we regrouped the industries into manufacturing and service sectors. Participants were recruited through the authors’ professional and personal connections. A cover letter assured the confidentiality of participants’ responses, clarified that participation was voluntary, and explained the scope of the study. Before distributing questionnaires to participants, the HR Manager of every firm briefed team members and managers about our research work and its objectives. Also, confidentiality was guaranteed. After that, questionnaires were distributed to 491 employees in 98 firms. To mitigate common method variance (CMV), data collection occurred at two distinct measurement points [49,50]: Time 1, which encompassed the independent variable and moderator, and Time 2, encompassing the mediators and dependent variable. In total, we received 399 questionnaires from 80 different firms, yielding an 81.26% response rate. A comprehensive research questionnaire is provided in Appendix A.

The sample consisted of 82% male participants and 18% female participants. Of the respondents, 27% were between the ages of 20 and 25, 32% between 26 and 30, 23% between 31 and 35, 8% between 36 and 40, 6% between 41 and 45, and 4% over 45.

Among the respondents, 40% possessed a Bachelor’s Degree, 41% held a Master’s Degree, and 19% held an M.S./M.Phil. Degree. Of the respondents, 52% were unmarried, and 48% married. In terms of position, 12% of respondents were first-line employees, 68% were middle-level managers, and 20% were high-level managers.

### 3.2. Measurement

Participants evaluated all items using a 5-point Likert scale, which ranged from ‘1 = strongly disagree’ to ‘5 = strongly agree’.

Perceived HPWS: We adapted [51] a ten-item scale to measure perceived HPWS. In accordance with the research conducted by [52], this scale was originally developed by [53]. An example item is ‘This company offers training to improve the interpersonal skills of employees’. One item was dropped out due to poor loading of less than 0.50. Cronbach’s α value for perceived HPWS was 0.902.

Bootlegging behavior: We adapted [10] a five-item scale to measure bootlegging behavior. An example item is ‘In addition to my formal job schedule, I have the freedom to explore new business ideas that might be profitable’. One item was dropped out due to poor loading of less than 0.50. Cronbach’s α was 0.867 for bootlegging behavior.

Willingness to take risks: We adapted [54] a three-item scale to measure willingness to take risks, which was established by [55]. An example item is ‘I am a risk-taker when it comes to proposing new ideas for achieving beneficial outcomes for the organization’. Cronbach’s α value for willingness to take risks was 0.781.

Creative self-efficacy: To assess creative self-efficacy, we utilized a three-item scale originally developed by [56], as adopted by [57]. An example item is ‘I have confidence in my ability to solve problems creatively’. Cronbach’s α was found to be 0.762 for creative self-efficacy.

HRM system strength: To evaluate HRM system strength, we employed an eight-item scale developed by [58], as adopted by [59]. An example item is ‘In this organization, rewards are clearly related to performance.’ Cronbach’s α value for HRM system strength was 0.896.

### 3.3. Common Method Bias

In order to evaluate the presence of common method bias, we employed the Harman single-factor test, as described by [49]. In addition to exploratory factor analysis (EFA), an unrotated factor solution was applied, revealing that one factor accounted for 26.44% of the total variance. This suggests that the presence of common method bias was not a significant concern.

### 3.4. Analytical Strategy

There were six main stages of our analysis. First, we ruled out the presence of common method bias. Second, we conducted a confirmatory factor analysis to verify the factor structure of our focal constructs. Third, we ran ICC1, ICC2, and rwg (J) to check whether a multi-level analysis could be conducted or not. Fourth, we checked correlations among the variables used in the study. Fifth, we used the Hayes Process macro to test the hypotheses (H1, H2, and H3) that constitute a direct or mediation effect. Sixth, we analyzed the cross-level effect of moderation to test hypotheses (H4a and H4b) through hierarchical linear modeling (HLM) software version 8.2.

## 4. Data Analysis and Results

### 4.1. Validity of Measures

To determine the discriminant validity, confirmatory factor analysis was performed by using R software (https://www.r-project.org/). Perceived HPWS, Bootlegging behavior, Willingness to take risks, Creative self-efficacy, and HRM system strength are the five latent variables in our research model. We conducted a comparison of fit between alternative one-, two-, three-, and four-factor models against our proposed five-factor model. The results, presented in Table 1, indicated that the five-factor model exhibited a more robust fit to the data in comparison to the alternative models. These findings provide evidence that all five variables under investigation in this study are indeed distinct from one another (see Table 1). Furthermore, Table 2 shows each variable’s reliability and validity value (see Table 2). The findings showed that the reliability and validity of the questionnaire are acceptable [60,61,62].

HRM system strength is an organizational-level variable. To validate the aggregation of individual members ratings at the organizational level, we calculated the within-group inter-rater agreement (rwg) [63] and interclass correlations (ICC1 and ICC2) [64]. We found that the average rwg (J) value for HRM system strength was 0.894, ICC1 was 0.307, and ICC2 was 0.994. In addition, we calculated ICC1 and ICC2 values for independent, dependent, and mediation variables. For perceived HPWS, ICC1 was 0.319, and ICC2 was 0.994. For bootlegging behavior, ICC1 was 0.330, and ICC2 was 0.994. For the willingness to take risks, ICC1 was 0.337, and ICC2 was 0.995. For creative self-efficacy, ICC1 was 0.284, and ICC2 was 0.993. Hence, ICC1 exceeds 0.05, which indicates that a multi-level study can be conducted.

### 4.2. Descriptives

Table 3 presents the means, standard deviations, and correlation coefficients for the variables under consideration. The findings indicate that perceived HPWS exhibited a positive association with bootlegging behavior, willingness to take risks, and creative self-efficacy. Additionally, bootlegging behavior demonstrated a positive relationship with willingness to take risks and creative self-efficacy (See Table 3).

### 4.3. Analysis of Direct and Mediation Effects at the Individual Level

Hayes process macro was used to test Hypotheses 1, 2, and 3 [65]. Hypothesis 1 proposes that perceived HPWS is positively associated with bootlegging behavior (see Table 4, direct effects). Hypothesis 2 suggests that willingness to take risks positively mediates perceived HPWS and bootlegging behavior. Hypothesis 3 proposes that creative self-efficacy positively mediates perceived HPWS and bootlegging behavior (see Table 4, mediation effects).

### 4.4. Analysis of Moderation Effect

**Figure 2:**
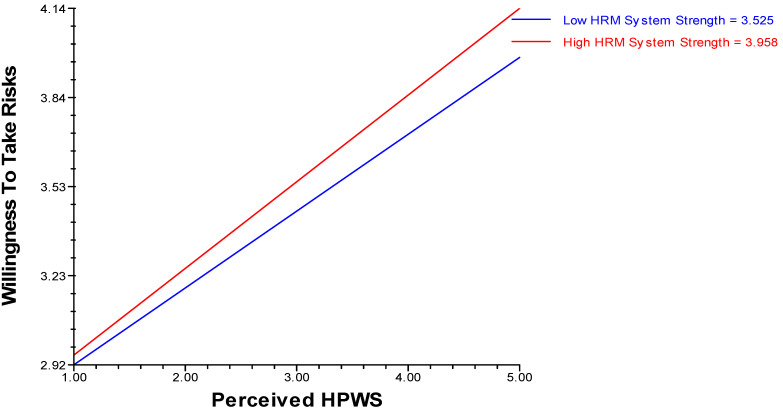
HRM system strength as a positive cross-level moderator in the relationship between perceived HPWS and willingness to take risks.

**Figure 3:**
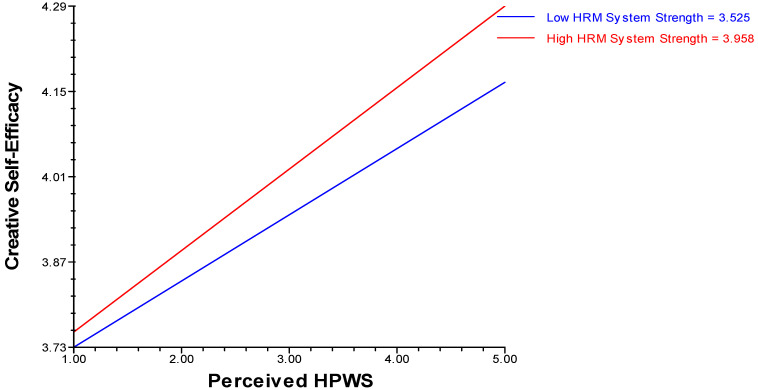
HRM system strength as a positive cross-level moderator in the relationship between perceived HPWS and creative self-efficacy.

Hypothesis 4 was tested using HLM software. Hypothesis (H4a) revealed that positive cross-level moderation of HRM system strength existed in relation to perceived HPWS with the willingness to take risks, whereas hypothesis (H4b) proposes that positive cross-level moderation of HRM system strength exists in relation to perceived HPWS with creative self-efficacy (see Table 5(a,b), moderation effects) (Figure 2 and Figure 3).

## 5. Robustness Check

**Figure 4:**
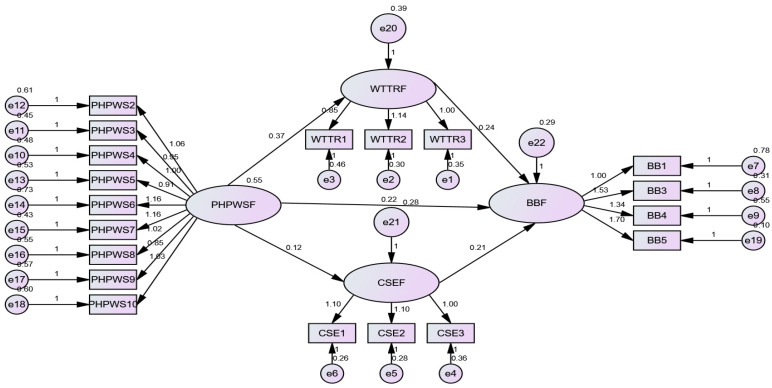
SEM robustness test results.

**Figure 5:**
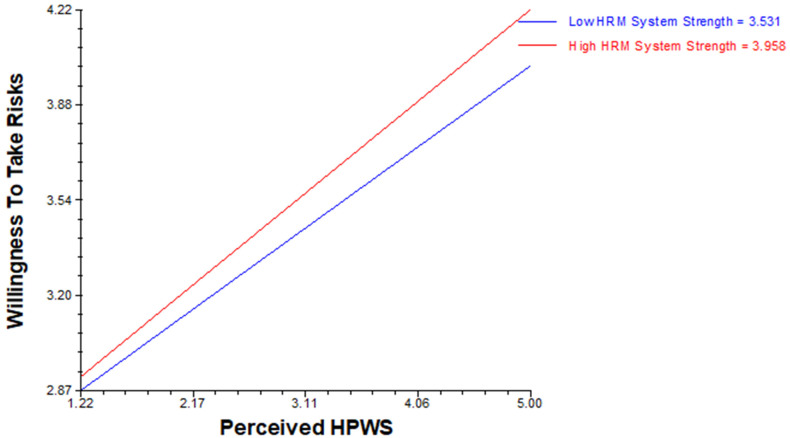
HRM system strength as a positive cross-level moderator in the relationship between perceived HPWS and willingness to take risks for robustness test.

**Figure 6:**
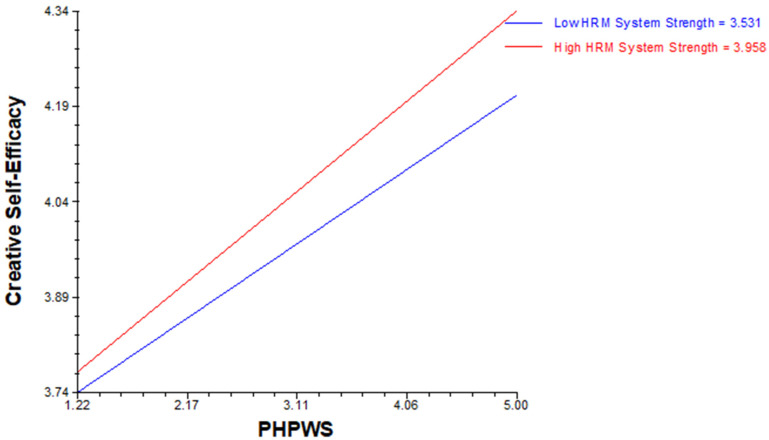
HRM system strength as a positive cross-level moderator in the relationship between perceived HPWS and creative self-efficacy for robustness test.

We also undertook a robustness check to ensure the credibility of the results. Specifically, as we used Hayes [65] PROCESS macro for SPSS to test our research hypotheses H1, H2, and H3, we employed a structural equation modeling (SEM) approach to test if the results were consistent (see Table 6, Figure 4). Moreover, we also checked the robustness of the results of hypotheses H1, H2, and H3 by adding the demographic control variables, including gender, age, qualification, marital status, and management levels (see Table 7). To test the robustness of cross-level moderation (H4a and H4b), we considered the impact of age on the studied relationship. We included this because participants’ status of indulging in bootlegging, taking risks, and belief in themself to perform creatively may be subject to their age. For example, junior employees may experience difficulty in managing their job demands. On the other hand, senior employees may be unable to focus because of old age [66]. Therefore, we excluded 10% of the sample (junior employees’ age 5% and senior employees’ age 5% and then tested the cross-level moderation by taking age as a control variable (see Table 8 and Table 9). The results of all robust tests as shown in Table 6, Table 7, Table 8 and Table 9 were significant, as before, without robustness tests (Figure 5 and Figure 6).

## 6. Discussion and Theoretical Contributions

This study explored the positive effect of perceived HPWS on bootlegging behavior. Results showed that perceived HPWS enhanced bootlegging behavior. Moreover, this research examined how and when willingness to take risks and creative self-efficacy exerted an effect on the connection between perceived HPWS and bootlegging behavior. Our results revealed that when employees perceived HPWS as intended, it enhanced their willingness to take risks and creative self-efficacy, which led to bootlegging behavior. Furthermore, our study investigated the cross-level moderating influence of HRM system strength on the association between perceived HPWS and both willingness to take risks and creative self-efficacy. Our findings revealed that when HRM system strength was higher, it amplified the relationship between perceived HPWS and both willingness to take risks and creative self-efficacy.

The study’s theoretical and literary contributions are reflected in three ways. First, we established a connection between perceived HPWS and the phenomenon of bootlegging, which pertains to bottom-up instructed outcomes, thus answering research calls to further examine the effect of perceived HPWS on a bottom-up instructed outcome like bootlegging. Previous research focused on the double-edged sword effect of perceived HPWS with top-down instructed outcomes [3,4,5,6,7,8]. We extend by concluding the positive effect of perceived HPWS on bootlegging behavior. Our findings also offer critical insight regarding the effect of perceived HPWS, e.g., HPWS does not always necessarily mean that it becomes the source of top-down instructed outcomes. HPWS can also become the source of bottom-up instructed outcomes, e.g., bootlegging behavior. To our knowledge, this study represents the first investigation of the positive effect of perceived HPWS on bootlegging behavior in the Pakistani context. The results also validated that perceived HPWS positively affects bootlegging behavior, thus enriching and extending social information processing theory.

Second, we incorporated willingness to take risks and creative self-efficacy as underlying mechanisms, thus answering research calls to further unravel the connection between perceived HPWS and bootlegging behavior. Prior researchers have looked at the mediators in the relationship between perceived HPWS and various outcomes [7,13,14,15,16,17]. We extended by exploring underlying mechanisms, e.g., willingness to take risks and creative self-efficacy, for a more nuanced understanding of perceived HPWS impact on bootlegging behavior. To our knowledge, this study is the first to indicate how willingness to take risks and creative self-efficacy mediate perceived HPWS and bootlegging behavior in the Pakistani context. The findings also confirmed that willingness to take risks and creative self-efficacy mediate the relationship between perceived HPWS and bootlegging behavior, thus enriching and extending social information processing theory.

Third, researchers examined how various moderators affect perceived HPWS and numerous outcomes [6,8,14,15,16,19]. To our knowledge, this study is the first to scrutinize HRM system strength as a cross-level moderator in the association between perceived HPWS and willingness to take risks and creative self-efficacy in the Pakistani context. The results also verified that perceived HPWS under the umbrella of high-level HRM system strength enhances willingness to take risks and creative self-efficacy, thus enriching and extending social information processing theory.

In summary, the theoretical and literary contributions of the present study are threefold. First, in light of prior research that has examined the positive and negative associations of perceived HPWS with diverse outcomes, this study presented a novel endeavor to illustrate the positive impact of perceived HPWS on bootlegging behavior. Second, the present study constituted a novel attempt to scrutinize attitudes, e.g., willingness to take risks and creative self-efficacy, as underlying mechanisms to comprehend the association between perceived HPWS and bootlegging behavior in depth. Third, following a comprehensive review of the literature pertaining to perceived HPWS, it is noteworthy that this study marks the inaugural exploration of the cross-level moderating influence of HRM system strength on the connection between perceived HPWS and both willingness to take risks and creative self-efficacy.

### 6.1. Practical Implications

This study carries practical significance for managerial implementation. This study highlighted the significance of HPWS in shaping employees’ perceptions, attitudes, and behaviors. Work practices such as recruitment, training, appraisal, rewards, and job security are valuable tools for managers to enhance employees’ willingness to take risks and creative self-efficacy, ultimately leading to improve bootlegging and other innovative behaviors. Management and HR can regulate such work practices. Managers can promote a culture of trust, sincerity, ethics, and self-confidence by fostering a strong association between willingness to take risks and creative self-efficacy for generating innovative behaviors like bootlegging. In addition, organizations and managers can foster a strong HRM system strength to create a positive atmosphere where employees can understand what is expected from them and what they will receive as a reward. By doing so, employees will be more likely to understand and perceive HPWS correctly, which could ultimately serve as a source to enhance willingness to take risks, creative self-efficacy, and innovative behaviors like bootlegging. In this way, organizations can achieve goals in an effective and efficient manner.

### 6.2. Limitations and Future Research Directions

Our research is subject to several limitations. First, our study only took into account perceived HPWS as an antecedent. Future research should take high-involvement work systems, high-commitment work practices, epistemic curiosity, empowering leadership, etc., as antecedents. Second, this study used HRM system strength as a moderator at an organizational level. Future research should focus on organizational climate, organizational strategy, and leadership style because these are also carriers of social information. Third, our study incorporated two mediators: willingness to take risks and creative self-efficacy. Research in the future could investigate the impact of perceived HPWS on various mediators after rigorously studying perceived HPWS literature to broaden the application of social information processing theory and effectively contribute to the literature. Fourth, the sample is entirely from Pakistan. Future research could consider other contexts to generalize the findings.

## Figures and Tables

**Table 1:** Results of confirmatory factor analysis.

Models	Factors	X^2^/df	RMSEA	SRMR	CFI	TLI
Model1	PHPWS + BLB + WTTR + CSE + HRMSS	9.809	0.149	0.156	0.386	0.433
Model2	PHPWS; BLB + WTTR + CSE + HRMSS	7.779	0.130	0.155	0.565	0.527
Model3	PHPWS; BLB + WTTR; CSE + HRMSS	3.412	0.078	0.083	0.846	0.832
Model4	PHPWS; BLB + WTTR; CSE; HRMSS	2.470	0.061	0.061	0.907	0.897
Model5	PHPWS; BLB; WTTR; CSE; HRMSS	1.560	0.037	0.038	0.965	0.961

Note: *N* = 399; PHPWS, perceived high-performance work systems; BLB, bootlegging behavior; WTTR, willingness to take risks; CSE, creative self-efficacy; HRMSS, human resource management system strength.

**Table 2:** Convergent and discriminant validity of scales.

Variables	CR	AVE
PHPWS	0.892	0.508
BLB	0.851	0.642
WTTR	0.814	0.551
CSE	0.838	0.519
HRMSS	0.916	0.526

Note: *N* = 399; PHPWS, perceived high-performance work systems; BLB, bootlegging behavior; WTTR, willingness to take risks; CSE, creative self-efficacy; HRMSS, human resource management system strength; AVE, average variance extracted; CR, composite reliability.

**Table 3:** Mean, standard deviations, and correlations.

Variables	Mean	SD	(1)	(2)	(3)	(4)	(5)	(6)	(7)	(8)	(9)	(10)
Gender (1)	1.177	0.382	1.00									
Age (2)	2.448	1.340	−0.127 *	1.000								
Qualification (3)	1.789	0.737	0.089	0.297 **	1.000							
Marital Status (4)	1.476	0.500	−0.181 **	0.667 **	0.232 **	1.000						
Management Level (5)	1.932	0.565	−0.025	−0.199 **	−0.167 **	−0.117 *	1.000					
Perceived HPWS (6)	3.386	0.795	0.001	0.055	−0.027	0.030	−0.014	1.000				
Bootlegging behavior (7)	3.334	0.937	0.016	−0.014	−0.061	−0.061	−0.041	0.386 **	1.000			
Willingness to take risks (8)	3.588	0.765	−0.038	−0.005	0.000	−0.027	−0.063	0.328 **	0.408 **	1.000		
Creative self-efficacy (9)	4.040	0.649	−0.046	0.028	0.054	−0.043	−0.043	0.127 **	0.229 **	0.198 **	1.000	
HRM system strength (10)	3.730	0.680	−0.026	−0.086	−0.021	−0.097	−0.051	0.224 **	0.117 *	0.160 **	0.153 **	1.000

Note: *N* = 399, ** *p* < 0.01, * *p* < 0.05.

**Table 4:** Analysis of direct and mediation effects without control variables.

Hypothesis	Path	Direct Effect	LL 95CI	UL 95CI
Direct effects	H1: Perceived HPWS → Bootlegging behavior	0.321	0.214	0.428
Mediation effects	H2: Perceived HPWS → Willingness to take risks → Bootlegging behavior	0.112	0.062	0.168
	H3: Perceived HPWS → Creative self-efficacy → Bootlegging behavior	0.020	0.002	0.046

Note: *N* = 399.

**Table 5:** (**a**) Cross-level moderation. (**b**) Cross-level moderation.

(**a**)	
**Willingness to take risks**	
Fixed Effect	
Willingness to take risks mean (β_0_)	
Intercept (γ_00_)	2.662 ***
Willingness to take risks slope (β_1_)	
Intercept (γ_10_)	−0.009
HRM System Strength (γ_11_)	0.077 *
Random Effects (Variance)	
Between	
HRM system strength (τ_00_)	1.127
HRM system strength (τ_11_)	0.086
Within	
δ^2^	0.442
Deviance	870.877
(**b**)	
**Creative self-efficacy**	
Fixed Effect	
Creative self-efficacy mean (β_0_)	
Intercept (γ_00_)	3.616 ***
Creative self-efficacy slope (β_1_)	
Intercept (γ_10_)	−0.096
HRM System Strength (γ_11_)	0.058 *
Random Effects (Variance)	
Between	
HRM system strength (τ_00_)	0.923 **
HRM system strength (τ_11_)	0.058 *
Within	
δ^2^	0.355
Deviance	779.160

Note: *N* = 399, *** *p* < 0.001, ** *p* < 0.01, * *p* < 0.05.

**Table 6:** SEM robustness test results.

Relationships	Estimate	S.E.	C.R.	*p*
PHPWSF → BBF	0.222	0.050	4.408	***
PHPWSF → WTTRF	0.373	0.057	6.578	***
WTTRF → BBF	0.242	0.057	4.251	***
PHPWSF → CSEF	0.116	0.044	2.664	0.008
CSEF → BBF	0.214	0.066	3.246	0.001

Note: *N* = 399, *** *p* < 0.001.

**Table 7:** Analysis of direct and mediation effects with control variables.

Hypothesis	Path	Direct Effect	LL 95CI	UL 95CI
**Control variables**	Gender → Bootlegging behavior	0.016	−0.212	0.245
	Age → Bootlegging behavior	0.017	−0.070	0.105
	Qualification → Bootlegging behavior	−0.060	−0.183	0.063
	Marital status → Bootlegging behavior	−0.156	−0.387	0.075
	Management levels → Bootlegging behavior	−0.080	−0.235	0.074
**Direct effects**	H1: Perceived HPWS → Bootlegging behavior	0.319	0.212	0.427
**Mediation effects**	H2: Perceived HPWS → Willingness to take risks → Bootlegging behavior	0.112	0.062	0.173
	H3: Perceived HPWS → Creative self-efficacy → Bootlegging behavior	0.020	0.002	0.047

Note: *N* = 399.

**Table 8:** Cross-level moderation for willingness to take risks.

Willingness to Take Risks	
Fixed Effect	
Willingness to take risks mean (β_0_)	
Intercept (γ_00_)	2.573 ***
Willingness to take risks slope (β_1_)	
Intercept (γ_10_)	−0.034
Perceived HPWS (β_2_)	
Intercept (γ_20_)	−0.025
HRM System Strength (γ_21_)	0.093 **
Random Effects (Variance)	
Between	
Intercept (τ_00_)	1.840
Age slope (τ_11_)	0.025
HRM system strength slope (τ_12_)	0.147
Within	
δ^2^	0.399
Deviance	770.718

Note: *N* = 399, *** *p* < 0.001, ** *p* < 0.01.

**Table 9:** Cross-level moderation for creative self-efficacy.

Creative Self-Efficacy	
Fixed Effect	
Creative self-efficacy mean (β_0_)	
Intercept (γ_00_)	3.601 ***
Creative self-efficacy slope (β_1_)	
Intercept (γ_10_)	−0.003
Perceived HPWS (β_2_)	
Intercept (γ_20_)	−0.093
HRM System Strength (γ_21_)	0.061 *
Random Effects (Variance)	
Between	
Intercept (τ_00_)	1.679
Age slope (τ_11_)	0.020
HRM system strength slope (τ_12_)	0.061
Within	
δ^2^	0.340
Deviance	689.331

Note: *N* = 399, *** *p* < 0.001, * *p* < 0.05.

## Data Availability

The data that support the results of the present study will be available from the corresponding author upon request.

## Appendix A

**Items**
HPWS
1. This company offers training to improve the interpersonal skills of employees.
2. New employees undergo extensive orientation training in order to learn the values and culture of the company.
3. Many of this company’s employees are moved through a series of different job assignments in order to prepare them for future assignments.
4. The employee selection process is very rigorous in this company (e.g., use of tests, aptitude tests, interviews, etc.).
5. There is advance planning as to which of this company’s current employees will be transferred or promoted when there is a job vacancy.
6. An employee’s job performance is appraised, to a significant extent, on how well he or she follows orders and company procedures and rules.
7. We strive to keep large salary differences between high and low performers in the same position.
8. An employee’s pay is closely tied to individual or group performance in this company.
9. Employees often work in self-directed teams.
10. This company extensively shares its financial and/or performance data with its employees.
Bootlegging behavior
1. In addition to my formal job schedule, I have the freedom to explore new business ideas that might be profitable.
2. My work plan does not allow me the time to work on anything other than the projects I have been assigned to.
3. I enjoy tinkering around with ideas that are outside the main projects I work on.
4. I am running several pet projects that allow me to learn about new areas.
5. I proactively take time to work on unofficial projects to seed future official projects.
Willingness to take risks
1. I like to play it safe when I am developing ideas to market this product.
2. I am a risk-taker when it comes to proposing ideas to market this product.
3. I prefer to think conservatively when I develop ideas for this product’s marketing program.
Creative self-efficacy
1. I have confidence in my ability to solve problems creatively.
2. I feel that I am good at generating novel ideas.
3. I have a knack for further developing the ideas of others.
HRM system strength
1. In this organization, rewards are clearly related to performance.
2. In this organization, the results of the yearly appraisals are generally considered to be fair.
3. HR staff have enough authority to get their ideas accepted.
4. In this organization, HRM is synonymous with excellent work.
5. The HR practices implemented in this organization sound good in theory but do not work in practice.
6. The appraisal procedure developed by the HR department has, in practice, effects other than the intended ones (Reverse coded).
7. Top management and HR management clearly share the same vision.
8. Management unanimously supports HR policy in this organization.
